# Presence of *Toxoplasma gondii* infection in brain as a potential cause of risky behavior: a report of 102 autopsy cases

**DOI:** 10.1007/s10096-018-3427-z

**Published:** 2018-11-23

**Authors:** Dorota Samojłowicz, Joanna Twarowska-Małczyńska, Aleksandra Borowska-Solonynko, Łukasz A. Poniatowski, Nipika Sharma, Mieszko Olczak

**Affiliations:** 1 Department of Forensic Medicine, Center for Biostructure Research, Medical University of Warsaw, Oczki 1, 02-007 Warsaw, Poland; 2 Department of General Biology and Parasitology, Center of Biostructure Research, Medical University of Warsaw, Chałubińskiego 5, 02-004 Warsaw, Poland; 3 Department of Experimental and Clinical Pharmacology, Centre for Preclinical Research and Technology (CePT), Medical University of Warsaw, Banacha 1B, 02-097 Warsaw, Poland; 4 Department of Neurosurgery, Maria Skłodowska-Curie Memorial Cancer Center and Institute of Oncology, W. K. Roentgena 5, 02-781 Warsaw, Poland; 5 Warsaw, Poland

**Keywords:** *Toxoplasma gondii*, Toxoplasmosis, Brain, Risky behavior, Alcohol, Mental health, Drivers

## Abstract

Toxoplasmosis was linked to impairment in brain function, encompassing a wide range of behavioral and neuropsychiatric changes. Currently, the precise localization of *Toxoplasma gondii* in the human brain is limited and the parasite DNA was not found in population-based screening of autopsy cases. The aim of proposed study was to identify the presence of parasite DNA within the brain and its association with risky behavior and alcohol consumption in postmortem examination. Preliminarily, 102 cases with certain circumstances of death at time of forensic autopsy was included. Due to high risk of bias, the females were excluded from the analysis and final study group consists 97 cases divided into three groups: risky behavior, inconclusively risky behavior, and control group. The obtained tissue samples for Nested PCR covered four regions of the brain: symmetric left/right and anterior/posterior horns of lateral ventricles comprising lining ependyma and hippocampus. The second type of material comprised blood evaluated for antibodies prevalence using ELISA and alcohol concentration using HS-GC-FID. Analysis demonstrated 16.5% prevalence concerning the parasite DNA presence in examined brain tissue samples without specific distribution and association with age at death or days after death until an autopsy was performed. Results have shown correlation between occurrence of risky behavior leading to death and higher proportions of positive parasite DNA presence within the brain. Correlation was not observed between parasite DNA presence and excessive alcohol consumption. Conducted screening demonstrated correlation between parasite DNA presence in the brain with risky behavior and provided new information on possible effects of latent toxoplasmosis.

## Introduction

*Toxoplasma gondii* is a common heteroxenous and polyxenous protozoan parasite which is globally widespread causing infection in virtually most warm-blooded mammal species, including humans [1, 2]. This remarkable virulence capacity and spread results from the ideal adaptation and complex function within the host-parasite axis, as well as the parasite’s high transmission potential [3]. In humans, parasite infection are related with the development one of an omnipresent systemic anthropozoonose referred to toxoplasmosis [4]. The epidemiological data shows that *T. gondii* infection affects almost all populations and demographic levels depending on a range of factors, including geographic region, seasonal changes, sanitary-hygienic conditions, socioeconomic status, and dietary habits [5, 6]. In general, approximately ~ 30% of the worldwide human population is infected by *T. gondii*, and the global prevalence is estimated to be in the range of 10–80% consisting a substantial public health problem [6, 7]. For a long time, according to previous published studies, the most common latent form of disease in immunocompetent subjects including pregnant women was thought to be asymptomatic and clinically normal [8]. Nevertheless, recent studies and observations from both human and preclinical animal models clearly indicate that this form of disease can exert formerly unrecognized short- and long-lasting negative consequences even in immunocompetent subjects [8, 9]. In particular, latent *T. gondii* infection in human populations was potentially linked to impairment in brain function, encompassing a wide range of behavioral and neuropsychiatric changes [10]. Among the behavioral domains, it was observed that latent *T. gondii* infection may trigger changes in impulsivity control including an increase in general risk-taking behaviors and violence where seropositive female individuals were found to exhibit increased levels of aggression, while male individuals were characterized by excessive impulsiveness [11]. Furthermore in regard to neurocognitive function, the latent infection was connected with psychomotor deficits, lack of concentration, lower intelligence quotient (IQ), and alternations in personality profile [12–14]. Collectively, these attributes may affect personality phenotype and could ultimately predispose to various life-threatening and health-risk behaviors, including suicide and homicide [15, 16]. Insights afforded from the last two decades also show potential link between latent *T. gondii* infection and increased risk of schizophrenia, depression, generalized anxiety, and obsessive-compulsive disease [17, 18]. Despite constant progress in both basic and clinical research, the current understanding of *T. gondii* action and influence mechanism in the human organism as an intermediate host is still limited [19]. The observed cerebral alternations consist a potential result of direct presence of *T. gondii* in different neuroanatomical locations within brain parenchyma [20]. Dopamine (DA) is an important catecholamine neurotransmitter and regulator which maintains stability and flexibility in a number of functions that include, but are not limited to cognition, locomotion, affect, reward, emotional, and neuroendocrine aspects [21, 22]. Reported observations from several studies support the concept that *T. gondii* infection followed by a local presence of parasite alters the concentration of neurotransmitters in the brain revealing one of the potential pathomechanisms leading to development of behavioral and neuropsychiatric changes by modulation of DA and its metabolite levels [23]. Another potential multiphasic event which contributes to changes in brain function includes local and systemic inflammatory reactions in response to the transposition and spread of *T. gondii* infection through the body [24, 25]. The direct presence of *T. gondii* within brain parenchyma is associated with secretion of inflammatory cytokines, chemokines, and mediators by neurons, astrocytes, and microglia, along with activation and recruitment of immune cells [26]. Persistent local neuroinflammatory reaction seems to also be related with alternations of neurotransmitter release [27]. The effect of *T. gondii* influence on the behavior of the intermediate host may be associated with the location of the parasite in specific regions of the brain, most likely those that are associated with fear and anxiety, mainly within the amygdala [28]. Another study indicated an increased number of parasites in the cerebral cortex, diencephalon, and hippocampus [29]. Despite these findings, the data concerning location of the parasite in the brains of a population with toxoplasmosis is not well investigated. In the one study conducted to date, in which *T. gondii* genetic material presence was evaluated by the Nested PCR method postmortem from the frontal cortex of psychiatric patients diagnosed during lifetime, the parasite DNA was not isolated and detected [30]. Given the evidence linking parasite infection to several behavioral and neuropsychiatric changes, the Department of Forensic Medicine at Medical University of Warsaw in collaboration with other academic departments of the university conducted extensive studies in an attempt to evaluate the precise identification of *T. gondii* DNA within the human brain and its association with risky behavior and alcohol consumption occurrence among an analyzed population in postmortem examination. Since this potential correlation has not been presented yet in the available literature, the results of the abovementioned studies pertaining to a correlation between *T. gondii* infection and risky behavior along with alcohol consumption have been presented in this manuscript.

## Materials and methods

### Autopsy cases

The study was carried out on samples obtained during external forensic examinations and autopsies performed and provided by forensic pathologists from the Department of Forensic Medicine at the Medical University of Warsaw over the 3-year period between 2010 and 2013. Cases included in the study covered only those whose circumstances surrounding the death were already known at the time of forensic autopsy or were previously provided by the appropriate Prosecutor’s Offices investigating the respective cases. Bodies presenting late postmortem signs as well as cases with incomplete medico-legal data were excluded from the study and did not undergo any further experimental testing. Based on external forensic examination and autopsy protocols along with data provided by the Prosecutor’s Office or in case of inpatients supplied medical records for each decedent, we collected the respective data: sex, age, circumstances of death, cause of death, period between death and autopsy, and (if provided) alcohol and/or illicit drug abuse status or the presence of psychiatric disorder. In the cases of traumatic deaths, the presence of brain injuries was also noted. Collectively, preliminarily a total of 102 cases (97 males and 5 females) were included in this study. The considerable ratio of male cases vs. female cases in the study sample was in accordance with the general proportion represented in all performed forensic autopsies in Department of Forensic Medicine at the Medical University of Warsaw, wherein nearly (~ 80%) of all autopsies the decedent is male. Consecutively, due to the high risk of bias, the females were excluded from the analysis and final study group consists 97 cases (*n* = 97). The estimated median age for the study group was 49 years (with the youngest case being 18 years and the oldest 89 years). The time period between death and autopsy ranged from 1 to 10 days with mean of 4.14 days. After collecting of autopsy reports and medico-legal data including additional tests, the circumstances of death were reviewed and then all evaluated cases were divided into three consecutive groups: risky behavior (RB), inconclusively risky behavior (IRB), and control (C) group. According to this, the RB group (*n* = 42) constituted cases wherein the available data indicated that the death was due to the so-called risky behaviors. These included drivers who were classified as indisputable perpetrators of traffic accidents (*n* = 35), individuals who died as a result of substance overdose (*n* = 5), and other individuals who died as a result of disregarding reasonable safety precautions (*n* = 2), where the first died of carbon monoxide (CO) poisoning and the second was a pedestrian. The cases whose deaths could not be precisely attributed to risky behaviors after reviewing all available data were included in the IRB group (*n* = 27). These included drivers who could not be clearly identified as the perpetrators of the traffic accident that caused their death (*n* = 14) and individuals who died as a result of disease whose cause could be reasonably suspected to be associated with chronic long-term alcohol consumption, although this fact was not verified (*n* = 13). The C group (*n* = 28) consisted of individuals whose deaths were not associated with risky behaviors, and who mostly died as a result of chronic disease.

### Tissue dissection and preparation

During the forensic autopsy, two types of material for examination were collected. The first type consisted of brain tissue samples which were obtained for genetic testing, while the second sample type comprised of blood used for serological testing. The brain specimens in a volume of approximately ~ 50 ml were collected to sterile tubes from four distinctive surrounding regions of brain: the symmetric left (A) and right (B) anterior horn of lateral ventricle comprising lining ependyma along with the symmetric left (C) and right (D) posterior horn of lateral ventricle comprising lining ependyma and hippocampus. Collection of brain tissue was excluded from areas comprising macroscopic signs of injury such as brain contusion and laceration, extra- and subdural hematoma, subarachnoid hemorrhage or intraventricular bleeding. Immediately after the dissection, brain tissue samples were stored at the ice cubes and kept away from lights following its safety transfer to the laboratory where the material were consecutively persevered at − 20 °C in special storage until the time of the preheating step and proper analysis. The blood samples (~ 10 ml) were collected directly from the cranial cavity or femoral vein into sterile tubes. After then, samples were centrifuged and stored at − 20 °C until the time of the test. The blood test was not conducted in three cases (male drivers) from the RB group due to exsanguinations of the bodies causing lack of material.

### Nested PCR

#### Initial digestion

Previously obtained and stored brain tissue samples were thawed at room temperature (20–24 °C) and then drained of residual blood. Brain tissue were homogenized, poured with a warm (37 °C) solution of 0.25% trypsin lyophilizate (3 U/ml) from bovine pancreas (Sigma-Aldrich, St. Louis, USA) which was previously dissolved in 0.9% saline (NaCl) solution and then digested in an oven at 37 °C for 1 h. During digestion the samples were mixed every 5 min. Afterwards, digestion samples were poured into 50-ml tubes and centrifuged at 400×*g* for 10 min. The supernatant was removed and then the sediment was washed twice in 0.9% NaCl followed by its centrifugation. In order to increase the efficiency of the method, the precipitate obtained after the initial digestion was equally distributed to three 1.5-ml tubes. In this way, three digestion products were obtained corresponding to each of the collected brain tissue sample. Collectively, a total of 12 DNA digestion products were obtained from one examined case.

#### Isolation of DNA

Isolation of DNA was carried out using the NucleoSpin Tissue Kit (740952; Macherey-Nagel, Düren, Germany) according to the manufacturer’s standard protocol. The precipitate was suspended in 210 μl of buffer T1 and 30 μl of proteinase K, and then was incubated for 24 h in thermomixer at 56 °C. When the initial incubation was done, buffer B3 was added in a volume of 230 μl and incubated for 10 min in thermomixer at 70 °C. After this time, 160 μl of 99.8% ethanol was added to the samples and the material was transferred to the spin column and centrifuged at 12,000 rpm for 5 min. The filtrate was removed and 500 μl of buffer BW was added to the column and centrifuged at 12,000 rpm for 5 min. After then, the filtrate was again removed, and 600 μl of buffer B5 was added to the column and centrifuged at 12,000 rpm for 5 min. The filtrate removal was repeated and 70 μl of buffer BE which was warmed to 70 °C and incubated for 1 min in room temperature was added to the column and then centrifuged at 12,000 rpm for 3 min. In this way, the obtained DNA isolates were stored at − 20 °C until the time of further use. In each case, isolation control was performed simultaneously. The sample was incubated and centrifuged under the same conditions and with the same reagents as the test samples, without the addition of sediment from the initial digestion.

#### Amplification of DNA

In order to detect the DNA of the *T. gondii* in the tested samples, a two-stage amplification was done. The initial amplification reaction was carried out using forward primer Tox4 (T1) and Tox5 (T2) reverse primer both covering 26 bp which attach to the end of the 3′ and to the 5′ end of the constitutively present fragment repeated 200–300-fold in the genome of *T. gondii* (Table 1). These oligonucleotide sequences were recognized as very specific and sensitive, and therefore remarkably useful in identifying of parasite. The primer sequences were designed in accordance with well-characterized and established available literature data [31]. The amplification reaction of *T. gondii* DNA was carried out using Taq PCR Core Kit (201223; Qiagen, Hilden, Germany) according to the manufacturer’s standard protocol. The amplification reaction mixture (50 μl) contained 5 mM MgCl_2_, 0.2 mM dNTP mix, 5 μl of PCR buffer (10×), 5 μl of CoralLoad PCR buffer (10×), 10 pM of each primer, 1 unit of Taq DNA polymerase, and 5 μl of DNA template where all was dissolved in sterile water purified from DNA nucleases. The PCR amplification was performed in a DNA thermal cycler (PTC-200; MJ Research, San Francisco, USA). Cycling conditions were as follows: initial denaturation step of 7 min at 94 °C, followed by 34 cycles of 1 min at 94 °C (denaturation), 30 s at 56 °C (primer binding), 30 s at 72 °C (elongation), and a final extension step at 72 °C for 10 min. After the reaction, a 529-bp product was obtained. Due to the inequivalent DNA concentration of the host and parasite in the analyzed samples, the Nested PCR method was used to detect *T. gondii* DNA in the obtained isolates. The sequence of the primers for the second amplification, as well as the optimal reaction conditions, was determined experimentally (Table 1). Subsequent amplification reaction of *T. gondii* DNA was carried out using the same reagents according to manufacturer’s standard protocol. Amplification reaction mixture (50 μl) contained 5 mM MgCl_2_, 0.2 mM dNTP mix, 5 μl of PCR buffer (10×), 5 μl of CoralLoad PCR buffer (10×), 10 pM of each primer, 1 unit of Taq DNA polymerase, and 0.5 μl of first amplification product where all was dissolved in sterile water purified from DNA nucleases. The subsequent PCR amplification was performed in a DNA thermal cycler (PTC-200; MJ Research, San Francisco, USA). Cycling conditions were as follows: initial denaturation step of 7 min at 94 °C, followed by 34 cycles of 1 min at 94 °C (denaturation), 20 s at 58 °C (primer binding), 20 s at 72 °C (elongation), and a final extension step at 72 °C for 10 min. After reaction, a 204-bp fragment contained in the first amplification product was obtained. During both amplification reactions, a positive and negative control was performed. In the first amplification reaction, the DNA isolated from the *T. gondii* culture was used for the positive control; in the second amplification, the control DNA from the first reaction was used for the positive control. In the negative control, pure water was added instead of DNA. The same reaction mixture as in the test samples was used in all controls.

**Table 1:** Primer sequences designed and used for first and second amplification of *Toxoplasma gondii* DNA

Primer	Primer sequences (5′-3′)	Predicted product size (bp)
T1	5′-CGCTGCAGGGAGGAAGACGAAAGTTG-3′	529
T2	5′-CGCTGCAGACACAGTGCATCTGGATT-3′	
T3	5′-GAGCCACAGAAGGGACAGA-3′	204
T4	5′-TTCCGGTGTCTCTTTTCCAC-3′	

#### Analysis of the products

The PCR products (10 μl) were separated and analyzed on a 2.0% agarose gel (Metaphor, FMC BioProducts, Rockland, USA) and visualized after staining with ethidium bromide. As a reference standard for DNA size marker, Gene Ruler 100 bp DNA Ladder Plus (MBI Fermentas, St. Leon-Rot, Germany) was used.

### ELISA

The serological prevalence of the *T. gondii* antibodies (IgG±) was evaluated using an enzyme-linked immunosorbent assay (ELISA) test. Followed assays were carried out using an ELISA test kit (Euroimmun, Lübeck, Germany) according to the manufacturer’s standard protocol. Reaction wells were added by following (100 ml) aliquots: calibration serum, positive control serum, negative control serum, and test serum samples diluted at 1:101. Each assay plate was covered and incubated at room temperature for 30 min. The wells were consecutively emptied and washed three times with 300 ml of diluted rinsing solution. Afterwards, anti-human IgG conjugated to peroxidase (100 ml) was added into each reaction well and incubated at room temperature for 30 min. The wells were washed three times after incubation. Substrate solution (100 ml) was added into each well and then incubated in a darkened area at room temperature for 15 min. The reaction termination was performed by adding 100-ml aliquots of a quench solution to each well in the same order and at the same speed as that used to add the substrate solution. Photometric assessment of color intensity at a wavelength of 450 nm (reference wavelength in the range 620–650 nm) was conducted using ELISA microplate reader (Ledetect 96; Dynamica, Salzburg, Austria) equipped with Micro Win 2000 software (Biochrom, Cambridge, UK). The interpretation of obtained results were done according to the instructions enclosed within the protocol. Each test was conducted in two subsequent repetitions.

### Analysis of alcohol levels

The alcohol (ethanol) concentration in obtained blood samples was analyzed using headspace gas chromatography-flame-ionization detection (HS-GC-FID) system (7090B; Agilent Technologies, Santa Clara, USA) coupled with a headspace sampler (7697A; Agilent Technologies, Santa Clara, USA). Two separated capillary columns were used including DB-ALC1 (30 m × 0.32 mm × 1.8 μm) and DB-ALC2 (30 m × 0.32 mm × 1.2 μm) column (Agilent Technologies, Santa Clara, USA). The incubation of samples was performed at 80 °C, the temperature of the transfer line was 110 °C, the temperature of the column was 40 °C, and the helium (He) carrier gas purity was 99.999% in a 5:1 split where the temperature of the detector was 300 °C. Ethanol levels were calculated from 100 μl of samples added to the 20-ml headspace glass vial and mixed with the 900 μl of internal standard solution (aqueous solution of 2-butanone). Vials were precisely closed and sealed with a rubber stopper. The obtained results from 90 cases (*n* = 90) were interpreted based on the definition of alcohol norms included in article 115 paragraph 16 point 1 of the Penal Code of the Republic of Poland, according to which individuals with a blood alcohol content equal to or exceeding 0.5‰ are considered to be under influence of alcohol.

### Statistical analysis

The statistical processing of the obtained data was performed using R statistical environment (version 3.4.3; R Development Core Team, Vienna, Austria). For group comparison analysis, Fisher’s exact test was used due to the limited number (*n* = 97) of available cases. In order to examine the hypothesis that *T. gondii* infection can be present in the brain and not in blood, the Monte Carlo simulation method was used. The detailed description of the method and the underlying principle is explained in the subsequent “Results” sections. All the obtained *p* values were corrected for multiple hypothesis testing using the *q* value algorithm. The results were considered statistically significant when *p* values were less than adjusted 0.05 (*p* < 0.05).

## Results

### Presence and distribution of the *T. gondii* DNA within brain

**Fig. 1:**
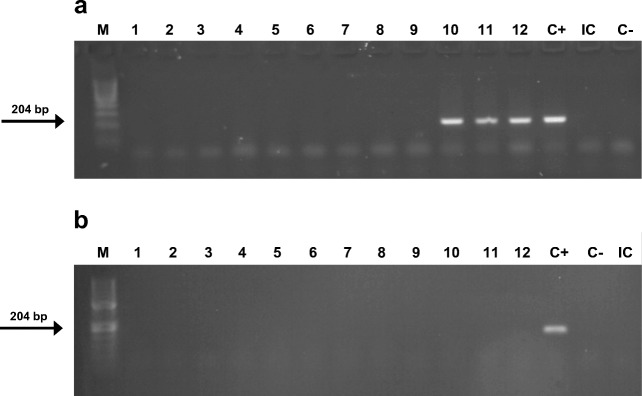
High-resolution agarose gel electrophoresis of Nested PCR products obtained from amplification of the brain tissue samples performed for detection of the *Toxoplasma gondii* DNA. **a** sample positive results from the single of evaluated cases. **b** sample negative results from the single of evaluated cases. The detailed reaction conditions are provided in the “Material and methods” section. M ladder, 1–12 lanes represent each of the PCR products obtained from the single of evaluated cases, C+ positive control, C− negative control, IC isolation control

**Fig. 2:**
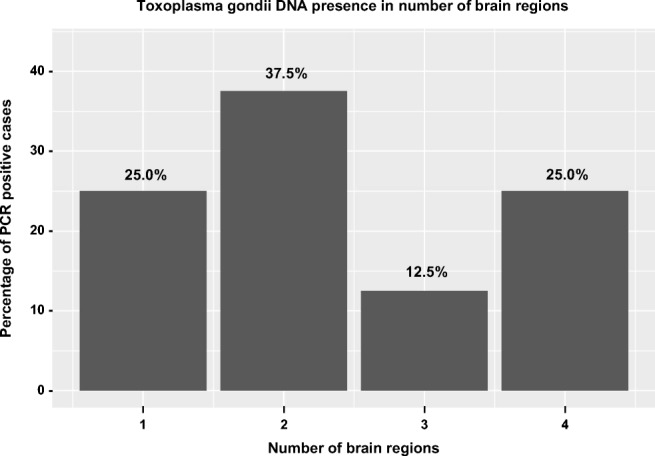
The schematic representation of the proportion of *Toxoplasma gondii* DNA presence in number of brain regions. PCR polymerase chain reaction

The presence of *T. gondii* DNA in the brain was detected in 16 (*n* = 16) out of 97 (*n* = 97) examined cases, which resulted in ~ 16.5% prevalence in our total studied population (Fig. 1). A subsequent analysis was made to determine in which of the four regions of the brain *T. gondii* DNA was most often detected. As was shown, in 12 (*n* = 12) out of 16 (*n* = 16) examined cases, the *T. gondii* DNA was detected in more than one region of the brain. *T. gondii* DNA was most frequently detected in samples taken from the B and C regions (Fig. 2). Since due to the number of positive cases was too small (*n* = 16), it is not possible to draw any statistically strong conclusions regarding brain localization of parasite cysts (Table 2).

**Table 2:** Location of the *Toxoplasma gondii* DNA presence in brain regions

	Brain region			
	A	B	C	D
Number of PCR positive cases	8	12	11	7
Percentage of PCR positive cases	50	75	68.8	43.8

A—left anterior horn of lateral ventricle comprising lining ependyma; B—right anterior horn of lateral ventricle comprising lining ependyma; C—left posterior horn of lateral ventricle comprising lining ependyma and hippocampus; D—right posterior horn of lateral ventricle comprising lining ependyma and hippocampus

### Serological prevalence of the *T. gondii* antibodies

The previously obtained positive test results for *T. gondii* DNA were confirmed (IgG+) serologically in ~ 56.3% of cases (*n* = 9). In ~ 18.7% of cases (*n* = 3), no serological tests were performed due to exsanguination of the respective bodies, causing lack of material. In other cases, the serological score was negative (IgG−). The 25% of cases (*n* = 4) in which the presence of the *T. gondii* DNA could not be confirmed serologically were found to be drivers (*n* = 2) who were classified as the indisputable perpetrators of traffic accidents from the RB group, a pedestrian (*n* = 1) from the RB group, and a driver (*n* = 1) who could not be clearly identified as the perpetrators of the traffic accident that caused their death from the IRB group. In addition, it is necessary to mention that in 75% of the above cases (*n* = 3), positive test results for *T. gondii* DNA were confirmed in ≥ 2 regions of the brain.

### Correlation between genetic and serological testing

The obtained results in the case of positive *T. gondii* DNA detection in the brain and negative serological antibody testing led to the proposal of a null hypothesis (H0): if serological testing results are negative, then genetic testing results are also negative. Given the nature of the relationship between genetic and serological testing, there are several plausible cases: *T. gondii* antibodies are not present in blood along with DNA in the brain (IgG−/DNA−), *T. gondii* antibodies are present in blood and DNA presence is confirmed in the brain (IgG+/DNA+), and *T. gondii* antibodies are present in blood while DNA presence is not confirmed in the brain (IgG+/DNA−). In order to determine whether the result that *T. gondii* DNA is present in the brain when antibodies are not present in blood (IgG−/DNA+), is statistically significant, the Monte Carlo simulation method was used. Since there were 97 cases (*n* = 97) of genetic positive results, 9999 random samples of size 97 were generated from the data. Given that there are 4 cases (*n* = 4) where *T. gondii* DNA was detected while serology was negative (out of 97 cases), the objective was to examine the number of times it is likely to get this or more extreme results if random samples from the data are generated. Assuming the significance level of 5%, we observed that the *p* value (*p* < 0.0086) was less than adjusted 0.05 in this case. Therefore, we rejected our null hypothesis that genetic testing will be negative if serological testing results are negative. Hence, we concluded that it is possible that *T. gondii* DNA is present in the brain even when antibodies are not present in the blood. In cases where *T. gondii* DNA is not present, both positive and negative results of serological testing were observed, with the percentage distribution of these results being similar, as well as a slight predominance of negative results (58% vs. 42%) in this case.

### Correlation between genetic testing and group comparison analysis

Subsequently, the relationship between *T. gondii* DNA presence and division including the RB, IRB, and C groups depending on medico-legal data which included additional tests as well as the circumstances of death was assessed (Table 3). According to this, among the RB group, test results for *T. gondii* DNA were positive in 11 (~ 11.3%) and negative in 31 (~ 32%) examined brain tissue samples. Out of 11 evaluated cases with a positive result from the RB group, 10 (~ 91%) died as a driver consisting the leading cause of death. In the IRB group, the 4 (~ 4. 1%) examined cases were positive, whereas 23 (~ 23.7%) were negative. Among the C group, 1 (~ 1%) examined case was positive with the remaining 27 (~ 27.8%) being negative results. Given these results, the *T. gondii* DNA presence within brain tissue in the RB group cases was higher than in cases of the IRB and C groups. In order to examine this relationship statistically, we used Fisher’s test of exact count because of reasons described above, i.e., due to limited number (*n* = 97) of available cases. The analysis showed that the difference in respect to the proportion of *T. gondii* DNA positive patients is significant among the groups (*p* = 0.0356). Afterwards, pairwise analysis was conducted in order to examine between which two groups the difference in the proportion of *T. gondii* DNA positive cases is significant. The comparison between the RB and C groups proportion showed a statistical relationship (*p* = 0.0209). We further confirm this association by calculating the odds ratio that is the odds of developing risky behavior when *T. gondii* infection is found in the brain, compared to odds of developing risky behavior in absence of it. Odds ratio of 3.55 establishes it is 3.55 times more likely to observe risky behavior in cases with positive test results for *T. gondii* DNA in the brain than without it. However, the correlation between the IRB and C group proportion showed no significant statistical relationship (*p* = 0.373). The comparison between the RB and IRB group proportion also showed no significant statistical relationship (*p* = 0.193).

**Table 3:** The proportion of individuals with positive and negative *Toxoplasma gondii* DNA presence test in brain stratified by groups

	Group		
	RB	IRB	C
Percentage of PCR positive cases	26.2	14.8	3.6
Percentage of PCR negative cases	73.8	85.2	96.4

*PCR* polymerase chain reaction, *RB* risky behavior group, *IRB* inconclusively risky behavior, *C* control group

### Correlation between genetic testing and time-dependent parameters

**Fig. 3:**
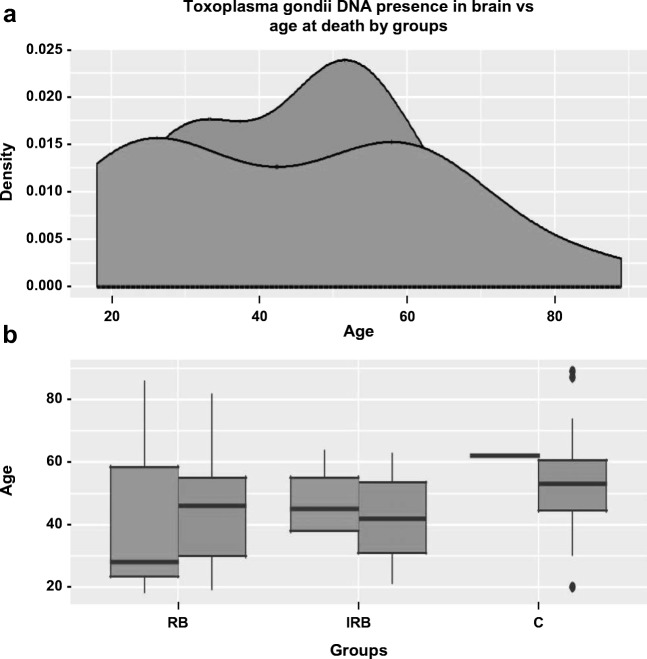
The proportion of individuals with positive and negative *Toxoplasma gondii* DNA presence test in brain stratified by age at death and groups. **a** Representation of the density function profile of cases by age. **b** Box plot representation. RB risky behavior group, IRB inconclusively risky behavior, C control group

No effect of *T. gondii* DNA presence in the brain on age at death was detected in our total studied population (Fig. 3). Subsequently, we conducted separate analyses for the RB, IRB, and C groups, but this also showed no evidence of a relationship between genetic testing and the age at death in any of the listed groups. There was also no relationship between the result of genetic testing and the number of days after death until an autopsy was performed and tissues were obtained. It is observed that the trends in number of days after death until an autopsy was performed were similar in both *T. gondii* DNA positive and negative individuals. Under both conditions, an autopsy was performed within ~ 5 days after death for most of the evaluated cases.

### Correlation between genetic testing and alcohol concentration

No relationship between *T. gondii* DNA presence in the brain and alcohol concentration in the obtained blood samples was detected in our total studied population (Table 4). A high *p* value clearly indicated that there is no dependence between *T. gondii* DNA presence and alcohol consumption in the overall data (*p* = 1.00). Diving into this analysis further, the relationship between *T. gondii* DNA presence and alcohol concentration was examined by segmenting the data using other variables. As it was presented, criteria by sex was unlikely to show any results, since there were no female subjects with positive *T. gondii* DNA presence test results. The performed tests showed that *T. gondii* DNA presence (*p* = 0.975) and age (*p* = 0.104) do not have any significant influence on alcohol consumption before death. The negative coefficient of age represents that the lower the age, the higher probability of dying drunk. The analysis does not show any significant relationship (*p* = 0.6891) between *T. gondii* DNA presence in the brain and cause of death as a driver after using alcohol.

**Table 4:** The proportion of individuals with positive and negative *Toxoplasma gondii* DNA presence test in brain stratified by detectable and undetectable alcohol concentration

	Alcohol concentration	
	≥ 0.5‰	≤ 0.5‰
Percentage of PCR positive cases	14.8	14.3
Percentage of PCR negative cases	85.2	85.7

*PCR* polymerase chain reaction

## Discussion

Despite considerable progress in basic and translational research concerning infectious diseases and their management, toxoplasmosis still consists a valid and challenging global problem [32]. This is directly related with the complex adaptable nature of this parasite, which is associated with its determinants of high virulence and transmission mode [33]. Insights afforded by ongoing studies and literature reports indicate the significance of the potential negative consequences of latent toxoplasmosis occurring in immunocompetent population, a group which was previously considered to be asymptomatic and uncomplicated [34]. Therefore, latent infection by *T. gondii* was linked to several diseases and states that impair the complex functioning of the brain [35]. As was mentioned before, these potential infection-induced behavioral and neuropsychiatric changes of the intermediate host are potentially a result of the direct well-targeted tropism of the parasite in specific neuroanatomical regions within the brain. According to the published observations concerning data obtained from preclinical in vitro and rodent models evaluating this issue, it was observed that parasite tissue cysts could be detected in all regions of the brain to a varying extent, with predilection to cortex, basal ganglia, amygdala, hippocampus, brain stem, and cerebellum [20, 28, 29]. However, in spite of these findings and knowledge that the brain is a main organ of encystment, the data concerning the location of parasite in the brain of people infected with toxoplasmosis is limited due to low proportion of neurones contain tissue cysts [35, 36]. This data comes mainly from histopathological studies evaluating the presence of toxoplasmosis in people who died of acquired immunodeficiency syndrome (AIDS) [37–39]. Despite compliance of animal and human studies on the location of parasite within brain of intermediate hosts, they did not provide sufficient and accurate information that would indicate one specific region in which the parasite cysts would be preferentially found. The above results indicate a rather random distribution of the parasite in various parts of the brain. In this regard, we have presented data documenting features concerning the expression of *T. gondii* DNA in cerebral tissue samples obtained from a population-based screening of autopsy cases. According to the available literature reports, this adopted methodology seems be an innovative approach, because to date, only one study was done, in which an attempt was made to examine the presence of *T. gondii* DNA in the frontal lobe of the brain in autopsy cases of population groups diagnosed with schizophrenia, bipolar disease, depression, psychosis, and affective disorder [30]. This attempt was unsuccessful, as in all of the examined cases a negative result was obtained. By general assumption, our study showed ~ 16.5% prevalence concerning the presence and expression of *T. gondii* DNA in the examined brain tissue samples from a population-based screening of autopsy cases. To our best knowledge, this is the first study published in available literature which successfully indicated the presence of *T. gondii* DNA within the brain in autopsy cases. We suppose that the initial digestion enabled us to search a larger volume of the brain tissue due to the increase concentration of parasite cysts after this process. We did not observe any specific distribution of *T. gondii* DNA among the examined brains where the B and C areas consists the most common regions where parasite genetic material was found. This type of methodology has limitations regarding the assignment of four brain regions (A–D) without precise indication of the structures and areas; this was done in order to increase the probability of finding *T. gondii* DNA within brain tissue due to previous unsuccessful attempts. Although the literature reports clearly indicate that there is a relationship between toxoplasmosis frequency and age, our collected data and performed analysis did not reveal any correlation between *T. gondii* DNA presence and time-dependent parameters including age at death [40]. This is, in all cases, consistent with the wide distribution of toxoplasmosis among all demographic levels [5]. However, according to the median age for the study group (49 years) with the youngest case being 18 years and the oldest 89 years, we also obtained consistent data regarding the fact that among population parasite, seroprevalence increases with age [5, 6]. Therefore, potential damage of the neuroanatomical structures and areas lining our designated regions is responsible for characteristic changes of brain morphology and function in populations with latent toxoplasmosis [41]. In regard to that specific issue, the correlation between *T. gondii* DNA presence within the brain and risky behavior was evaluated. Our analysis and obtained results clearly suggest that the occurrence of risky behavior positively correlates with higher proportions of positive *T. gondii* DNA presence within the brain. This is in accordance with previously conducted studies regarding the influence of toxoplasmosis on the induction and occurrence of behavioral and neuropsychiatric changes [8, 10, 42]. In reference to the results of previously published reports, the interpretation of direct parasite presence confirmed by its DNA detection and risky behavior relationships in population-based screenings of autopsy cases was first presented in our study and indicates that such a correlation exists. To date, many literature reports have been published that explain how the parasite can affect the behavior of the intermediate host [43]. One such potential contributing factor is the effect of inflammatory cytokines produced in response to chronic invasion of *T. gondii* in the brain [44, 45]. Inflammatory cytokines have been shown to influence the metabolism of tryptophan (TRP) leading to a decrease in serotonin (5-HT) levels [46]. Their effects include the increase of indoleamine 2,3-dioxygenase (IDO), which metabolizes TRP into kynurenine (KYN), causing its level reduction in serum and, as a consequence, that of 5-HT levels, whose deficiency plays a significant role in the pathomechanism of depression and other psychiatric disorders [47, 48]. Studies conducted in recent years have also shown that neurotropic agents such as *T. gondii* create alterations on the level of neurotransmitters in the brain, including DA levels [49]. Altered neural processes in which DA production, secretion, or action is abnormal lead to the development of several neuropsychiatric disorders that involve abnormal cognitive and affective function [50]. Elevated levels of DA in the brains of rodents with chronic infection of *T. gondii* were first noticed and described in 1985 on the mouse model [51]. It was found that the respective DA level is 14% higher when referring to the control group, while mice with acute infections also showed a 40% rise in homovanillic acid (HVA) without changes concerning DA levels. A similar relationship was also observed in populations with toxoplasmosis; the results presented indirect proof and were produced using the Temperament and Character Inventory (TCI) questionnaire in groups of blood donors and military personnel [14, 52]. It was shown that the study population infected with *T. gondii* received fewer points compared to the respective control group in this questionnaire. A lower number of points in the questionnaire could be potentially associated with an increased concentration of DA in the brain. These observations implied that there is a specific mechanism for the synthesis and packaging of DA mediated by the direct presence of *T. gondii* in the brain [53]. Another mechanism linked excessive production of DA in response to interleukin 2 (IL-2), released by leukocytes in infected parts of the brain where local inflammation occurred [54]. It was noticed that similar results to the TCI questionnaire was obtained by populations infected with cytomegalovirus (CMV) and herpesvirus (HHV), who also had neuroinflammatory foci in their brains. Nowadays, it is known that DA accumulates in brain cells containing *T. gondii* cysts, where it is produced and released [23]. It was observed that deregulation of DA levels by the parasite occurs through extracellular secretion of tyrosine hydroxylase (TH) which consists an enzyme involved in their synthesis. Analysis of the *T. gondii* genome shown that it contain two genes encoding TH [55]. It collectively seems that the alterations in DA levels in the brain of infected individuals leads to behavioral and neuropsychiatric changes, but the way it affects specific regions in the brain still needs further investigation. Another mechanism was presented in the study conducted by McConkey et al., based on the analysis of the pathway for DA biosynthesis [56]. The authors emphasize that although the TH gene was found in the genome of parasite, the synthesis of this neurotransmitter takes place not in one, but in two enzymatic reactions. Thus, one more enzyme such as aromatic l-amino acid decarboxylase (AADC) is necessary in this case to convert l-3,4-dihydroxyphenylalanine (l-DOPA) into DA in infected cells [57]. According to this study, the l-DOPA released from parasite cysts is secreted into host neurons and then converted into DA. Therefore, regardless of the location of the parasite cysts in the brain, DA is always produced in the same neurons. At present, the precise indication of the complex mechanism systems in which the parasite affects the host’s brain is needed, despite the literature indicating several possibilities of these interactions, including the inflammatory and neurochemical aspects. A more precise analysis of the individual types of risky behavior revealed that there were RB subgroups in which the proportion of positive *T. gondii* DNA presence was present, but was not statistically higher in the driver population. However, recently published observations concerning this task suggest a connection between toxoplasmosis and an increased risk of causing a traffic accident. In the retrospective study of Flegr et al. in a group of 146 cases (drivers and pedestrians who contributed to the road accident) compared with 446 cases from the control group, a statistically significant relationship between latent toxoplasmosis and the risk of road accidents was observed [58]. Drivers infected with toxoplasmosis also have a prolonged reaction time, and therefore cause more road accidents than healthy drivers. Further observations conducted by Kocazeybek et al. have also shown an increased risk of causing a car accident in the group of seropositive drivers, compared to the control group of drivers who have never had a road accident [59]. It should be remembered that a road accident often depends on many variables whose contribution can be divided into all of its participants [60]. Therefore, a result showing that almost all cases from the RB group consist of drivers with confirmed presence of *T. gondii* DNA within the brain is not an insignificant result in this case. The tropism of *T. gondii* within the brain and its associated influence on specific neuroanatomical regions may have an impact on the perception of the environment and could potentially contributes in traffic road accidents. Moreover, it should be stated that non-compliance with standards and disregarding rules is one of the characteristics characterizing people with toxoplasmosis [42]. This could lead to the conclusion that the violation of traffic road regulations, which are intended to guarantee public security, is the occurrence of some factor that changes the perception of reality and correct assessment of the situation on the road by drivers which could in this case be the presence of a *T. gondii* infection [61]. Simultaneously, such a conclusion should not be formulated in isolation from cultural conditions and customs prevailing among the studied population of drivers [62]. To date, the available literature reports did not provide a link between latent toxoplasmosis and alcohol consumption. However, we have previously shown a correlation between latent toxoplasmosis and excessive alcohol consumption; this was observed as a higher proportion of positive serological tests in the evaluated cases [63]. Surprisingly, unlike our previous results, our presently collected data and performed analysis did not reveal any correlation between *T. gondii* DNA presence within the brain and excessive alcohol consumption. According to this incompatibility between genetic and serological testing, it is important to resolve this issue by performing further detailed studies focused on this potential correlation, as it can have a potential impact in research concerning alcohol abuse prevention and treatment [64, 65]. It is worth noticing that there were cases in which the presence of *T. gondii* DNA was detected in the brain, but the serologic test was negative. Such a state of affairs can have various causes, from technical reasons related to work on the autopsy material, which always involves the possibility of influencing posthumous changes in the obtained results, to numerous pathophysiological reasons leading to the fact that the antibodies were not produced or their level decreased to low or undetectable values [66]. Due to the very small number of such cases in this study, unambiguous conclusions cannot be drawn, and this issue also requires further detailed research. Observations’ finding that *T. gondii* infection may be present despite the negative result of serological tests could have clinical significance, especially in cases associated with severe immunological disorders [67]. It is not possible to explicitly rule out the existence of immunological disorders in the studied population of the autopsy cases because we did not have full clinical data on their health status; this consisted the next limitation of our study. However, according to our collected data and performed analysis, we provided an important contribution and filled the gap in research on the effects of the direct presence of *T. gondii* within the brain, evaluated in a population-based screening of autopsy cases. Most of all, the genetic test results demonstrated a strong correlation between *T. gondii* DNA presence within the brain and engaging in risky behavior potentially leading to death. The widespread dissemination of toxoplasmosis around the entire globe, concerning the research carried out so far, indicates that *T. gondii* may contribute to hundreds of thousands of deaths worldwide, including deaths in road accidents, accidents at work, and suicides [68, 69]. Demonstration of the collective relationship between *T. gondii* infection and risky behavior would potentially indicate the need for regular screening among people performing responsible professions, such as pilots, air traffic controllers, or professional drivers [70]. Adequate treatment of infected people could possibly reduce the number of road accidents and suicides, as well as reduce the risk of developing neuropsychiatric disorders, all of which definitively deserves further attention.
